# A Hybrid Model to Predict Formulation Dependent Granule Growth in a Bi-Component Wet Granulation Process

**DOI:** 10.3390/pharmaceutics13122063

**Published:** 2021-12-02

**Authors:** Indu Muthancheri, Rohit Ramachandran

**Affiliations:** Department of Chemical and Biochemical Engineering, Rutgers, The State University of New Jersey, Piscataway, NJ 08854, USA; im225@scarletmail.rutgers.edu

**Keywords:** wet granulation, multicomponent, population balance model, content uniformity

## Abstract

In this study, a hybrid modeling framework was developed for predicting size distribution and content uniformity of granules in a bi-component wet granulation system with components of differing hydrophobicities. Two bi-component formulations, (1) ibuprofen-USP and micro-crystalline cellulose and (2) micronized acetaminophen and micro-crystalline cellulose, were used in this study. First, a random forest method was used for predicting the probability of nucleation mechanism (immersion and solid spread), depending upon the formulation hydrophobicity. The predicted nucleation mechanism probability is used to determine the aggregation rate as well as the initial particle distribution in the population balance model. The aggregation process was modeled as Type-I: Sticking aggregation and Type-II: Deformation driven aggregation. In Type-I, the capillary force dominant aggregation mechanism is represented by the particles sticking together without deformation. In the case of Type-II, the particle deformation causes an increase in the contact area, representing a viscous force dominant aggregation mechanism. The choice between Type-I and II aggregation is determined based on the difference in nucleation mechanism that is predicted using the random forest method. The model was optimized and validated using the granule content uniformity data and size distribution data obtained from the experimental studies. The proposed framework predicted content non-uniform behavior for formulations that favored immersion nucleation and uniform behavior for formulations that favored solid-spreading nucleation.

## 1. Introduction

The modeling of wet granulation process provides better insight into its process dynamics, which then can be used for efficient process control and scale-up if required [1,2]. The simultaneous, interacting mechanisms in the wet granulation process make it a difficult unit operation to model. Various techniques are used for the modeling of granulation (based on time scale and length scale). The population balance model (PBM) or PBM coupled with particle-level models such as discrete element method (DEM) and continuous fluid dynamics (CFD) are extensively used for this purpose [3,4,5]. Although PBM captures multi-dimensional properties of granules such as size, composition, and porosity, the model predictions are restricted to the experimental design space due to the large number of fitting parameters involved. DEM is a physics-based model that tracks the particle positions based on collisions between particles and therefore is not impacted by design space changes. However, DEM is unable to independently simulate particle property changes resulting from various mechanisms in the wet granulation process. A coupled or hybrid DEM-PBM is found to be useful for comprehensively representing wet granulation at all scales (micro-meso scale using DEM and meso-macro scale using PBM). The macro-scale models the overall particle property change, the meso-scale represents property change within a granule ensemble, and the micro-scale represents individual particle or constituent powder dynamics.

A significant number of modeling studies have been conducted for the prediction of a single-component wet granulation process, and this includes both nucleation and growth kinetics [6,7,8,9]. Although most of the wet granulation process involves more than one solid component, there are not many models reported that consider a multi-component process. Building a formulation-dependent constitutive equation requires the understanding of the effects of primary particle properties such as maximum pore saturation, material yield strength, cohesiveness, and primary particle size, on the final product granule attributes.

Matsoukas et al. [10,11] and Marshall Jr et al. [12] applied a multi-component PBM for modeling a bi-component aggregation using a composition-dependent aggregation kernel. In their work, two components, solute and solvent, are considered. The internal coordinates of the granule or particle were determined by the total mass of the granule and the mass of solute in it. In the Matsoukas et al. [10] model, the solute is soluble in the solvent, and the bivariate distribution of number density is shown as the product of the size distribution, with a Gaussian compositional distribution. In an insoluble system, the Gaussian compositional distribution function that is used to represent the relative concentration of one component to another may not be applicable. Matsoukas et al. [11] represented a size-dependent aggregation kernel with a composition-dependent multiplicative factor. They defined an adjustable interaction parameter that describes the attraction or repulsion between the two components. The model was able to demonstrate the extent of blending with positive and negative interaction parameters.

The granule growth (aggregation/coalescence), however, is expressed nearly always as a semi-empirically based [4,13] kernel or as a function of the particle collision information obtained from DEM modeling [14,15]. Some of the limitations of the existing coalescence models for representing granule growth are that they neglect capillary force interaction and use the static yield strength analyses of the powder bed to calculate the granule strength during the collision. A multi-dimensional population balance [16] that accounts for size, solid content, surface liquid, and deformability needs to be used to couple “aggregation” and “layering” granule growth mechanisms.

Another mechanism to consider is the nucleation process at the start of the liquid addition phase during granulation, that impacts the granule growth mechanism and the final granule quality attributes. There are two important aspects of nucleation modeling: the kinetics of nuclei formation and the physical attributes such as size, porosity, and content uniformity of the nuclei. In the case of immersion nucleation, kinetic models were developed by Hounslow et al. [17], Hapgood et al. [18]. These kinetic models provide the nuclei size distribution as model output and which can be used for predicting the final granule size distribution [19]. The time scale of the nucleation process is relatively faster than the rest of the granulation rate mechanisms [20], and thus, it is not necessary to incorporate the dynamics of the nucleation process into the granulation modeling. However, the internal properties of nuclei such as size, deformability, surface liquid content, and content uniformity affect the granulation growth kinetics, and the final granule quality attributes [21,22]. Thus, it is important to have an experimental or modeling framework to predict the properties of the initial nuclei to simulate granule properties other than size distribution using PBM.

Physics-based models for simulating nuclei require complicated multi-phase simulations. Washino et al. [23] presented a coupled DEM and constrained interpolation profile (CIP) for simulating the nuclei during the wet granulation process. The effect of surface tension on the liquid binder flow was modeled depending on the relative position of the fluid interfaces to the solid particles, i.e., the model on the outside, inside, or on the surface of the powder bed corresponds to a free surface, capillary action, and bed surface wetting. Washino et al. [24] showed the CFD-DEM simulation of nuclei generation in a dynamic powder bed. These studies consider a particle system with good wettability or spreading coefficient [25]. In the case of powder mixture with hydrophobic powders, the nuclei formed are solid-spread nuclei, and the physics-based approach developed for immersion nuclei is not suitable for such systems. Due to the complexities of nucleation mechanisms that can be either an immersion or solid-spread nuclei based on the wettability of the constituent material on the droplet vicinity during binder addition, the currently available models fall short of representing the nucleation mechanism and nuclei property in such systems.

Hybrid modeling has been demonstrated to have various advantages of improving process understanding with the incorporation of empirically based statistical models with mechanistic models [26,27,28]. A hybrid model consisting of both statistical correlations and physics-based models is often used to simplify the computational efforts and incorporate complex mechanisms into the model. Such models can overcome the disadvantages of both purely data-driven and physics-based models [27]. A hybrid model such as PBM with artificial neural network (ANN) was developed to substitute for the high-fidelity PBM-DEM model by Barrasso et al. [29].

The objectives of this study are as follows:

1. Incorporate the nuclei particle characteristics in the population balance model based on the classification model result from Muthancheri et al. [30].
2. Develop a composition-dependent PBM framework for bi-component wet granulation process with a large binder droplet for predicting the granule quality attributes with change in percentage formulation.

## 2. Model Development

### 2.1. Population Balance Model

The population balance equation as shown in Equations (1) and (3) are used in this work to predict the particle size distribution, liquid distribution, and component distribution or the content uniformity [31]. In this work, the liquid volume of the granules is considered to be a lumped parameter under the assumption that all granules of the same size with the same composition of solids and pore volume have the same average liquid content. Such a reduced-order model was compared with higher-order models and was reported to have a significant time saving without compromising much on accuracy in previous studies [32].

(1) $$\begin{array}{cc} \; & \frac{\partial F}{\partial t}+\frac{\partial }{\partial s_{1}}\left(F\frac{ds_{1}}{dt}\right)+\frac{\partial }{\partial s_{2}}\left(F\frac{ds_{2}}{dt}\right)+\frac{\partial }{\partial p}\left(F\frac{dp}{dt}\right)={ℜ}_{nuc}+{ℜ}_{agg}++{ℜ}_{brk} \end{array}$$

(2) $$\begin{array}{cc} \; & \frac{\partial L_{i}}{\partial t}={ℜ}_{nuc,l_{i}}+{ℜ}_{agg,l_{i}}+{ℜ}_{brk,l_{i}}+F\frac{dl_{i}}{dt} \end{array}$$

(3) $$\begin{array}{cc} \; & \frac{\partial L_{e}}{\partial t}={ℜ}_{nuc,l_{e}}+{ℜ}_{agg,l_{e}}+{ℜ}_{brk,l_{e}}+F\frac{dl_{e}}{dt} \end{array}$$

where $F=F(s_{1},s_{2},p,t)$ is the number of particles with API volume $s_{1}$ and excipient volume $s_{2}$, pore volume *p* at time *t*. $L_{e}=L_{e}\left(s_{1},s_{2},p,t\right)$ and $L_{i}=L_{i}\left(s_{1},s_{2},p,t\right)$ is the average external liquid volume and average internal liquid volume of particles with API volume $s_{1}$ and excipient volume $s_{2}$, pore volume *p* at time *t*, respectively. Table 1 represents the dependent and independent variables. The rate mechanisms $\frac{ds_{1}}{dt}$, $\frac{ds_{2}}{dt}$, $\frac{dl_{i}}{dt}$, $\frac{dl_{e}}{dt}$, ${ℜ}_{nuc}$, ${ℜ}_{agg}$, and ${ℜ}_{brk}$ are detailed in the next section.

The relationship between the variables in Table 1 are summarized in the following equations. The total granule volume *v* is obtained by Equation (4).

(4) $$\begin{array}{c} v=s_{1}+s_{2}+l_{e}+p \end{array}$$

The surface area of the particles can be derived from the granule total volume as shown in Equation (5)

(5) $$\begin{array}{c} a=\pi^{\frac{1}{3}}{\left(6v\right)}^{\frac{2}{3}} \end{array}$$

The porosity and content uniformity can be calculated using Equations (6) and (7), respectively.

(6) $$\begin{array}{cc} \epsilon & =\frac{p}{v} \end{array}$$

(7) $$\begin{array}{cc} q & =s_{1}/v \end{array}$$

### 2.2. Mechanisms Involved in the Model

The rate mechanisms during wet granulation determine the final characteristics of a granule. The following rate mechanisms are considered in the current model:

1. Immersion nucleation
2. Solid-spread nucleation of hydrophobic API
3. Granule surface wetting during liquid addition
4. Granule surface growth due to solid-spread nuclei
5. Hydrophilic excipient layering
6. Particle aggregation
7. Particle breakage
8. Compaction

#### 2.2.1. Immersion Nucleation

Immersion nucleation kinetics are assumed to be instantaneous, with the nuclei being formed as soon as the drop hits the powder bed. The number of immersion nuclei generated $N_{im}$ is calculated by Equation (8).

(8) $$\begin{array}{cc} N_{im} & =P_{im}\frac{{\dot{Q}}_{spray}\times \Delta t}{v_{d}} \end{array}$$

where $Q_{spray}$ is the volumetric spray rate of binder liquid and $v_{d}$ is the volume of a single drop calculated from the nozzle opening. $P_{im}$ is the probability of immersion nucleation to happen for the given percentage composition of API powder bed (calculated from the classification model from Muthancheri et al. [30]). All the immersion nuclei is assigned to the first bin of same percentage composition as that of the powder bed and pore volume close to the volume of single droplet.

The mass of API ($M_{s_{1}}$) and excipient ($M_{s_{2}}$) available is calculated at every time step based on the mass balance of nuclei generated and excipient particle layering. The volume of immersion nuclei ($v_{im}$) at the end of nucleation can be estimated from the equation derived by Hounslow et al. [17] (Equation (9)).

(9) $$\begin{array}{cc} v_{im} & =v_{d}\left(1+\frac{1-\phi_{cp}}{\phi_{cp}}\right) \end{array}$$

(10) $$\begin{array}{cc} v_{im,s} & =v_{d}\left(\frac{1-\phi_{cp}}{\phi_{cp}}\right) \end{array}$$

where $\phi_{cp}$ is the critical-packing liquid volume fraction, which is kept constant at 0.2 in this study. $v_{im,s}$ is the solid volume in the nuclei. Total mass of solid component $M_{im,si}$, where i=1,2 for API and excipient, respectively, utilized to form immersion nuclei at Δt can be calculated as follows:

(11) $$\begin{array}{cc} M_{im,si} & =f_{i}N_{im}m_{im,s} \end{array}$$

(12) $$\begin{array}{cc} \; & =f_{i}N_{im}\rho_{s}v_{im,s} \end{array}$$

where $m_{im,s}$ is the solid mass in a single immersion nuclei and $\rho_{s}$ is the weighted true density of the solid components. $f_{i}$ is the fraction of solid component present in the powder bed (i=1,2 for API and excipient, respectively).

#### 2.2.2. Solid-Spread Nucleation

In the current model, the hydrophobic API is considered to form solid-spread nuclei, and the number of solid-spread nuclei ($N_{ss}$) is calculated as shown in Equation (13).

(13) $$\begin{array}{cc} N_{ss} & =\left(1-P_{im}\right)\frac{{\dot{Q}}_{spray}\times \Delta t}{v_{d}} \end{array}$$

**Figure 1:**
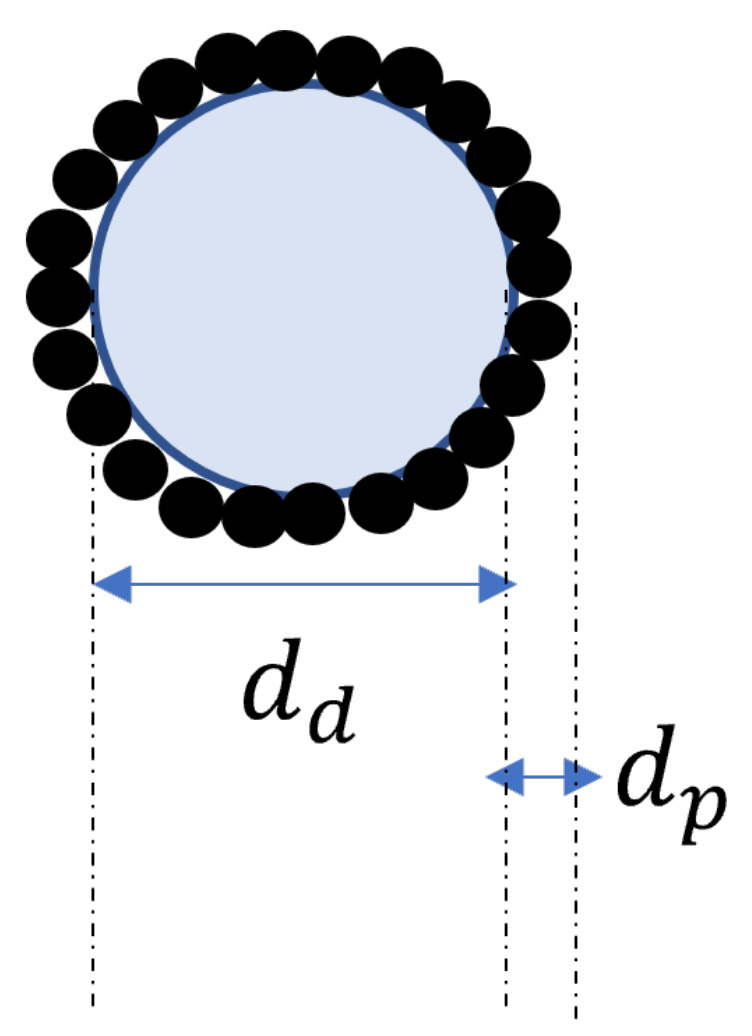
Solid-spread nuclei schematic.

Assuming that in the solid-spread nucleation the liquid drop (diameter $d_{d}$) is surrounded by hydrophobic API particles of diameter ($d_{p}$) (Figure 1), the approximate solid-spread nuclei volume ($v_{ss}$) and the volume of API particles in a solid-spread nuclei ($v_{ss,s1}$) can be calculated as follows:

(14) $$\begin{array}{cc} v_{ss} & =\frac{\pi }{6}{\left(d_{d}+2d_{p}\right)}^{3} \end{array}$$

(15) $$\begin{array}{cc} v_{ss,s1} & =\frac{\pi }{6}{\left(d_{d}+2d_{p}\right)}^{3}-v_{d} \end{array}$$

The total mass of API particles that form solid-spread nuclei at time Δt can be calculated as shown in Equation (17).

(16) $$\begin{array}{cc} M_{ss,s1} & =f_{1}N_{ss}m_{ss,s1} \end{array}$$

(17) $$\begin{array}{cc} \; & =f_{1}N_{ss}\rho_{s1}v_{ss,s1} \end{array}$$

where $m_{ss,s}$ is the solid mass in a single solid-spread nuclei and $\rho_{s1}$ is the weighted true density of the API.

#### 2.2.3. Granule Surface Wetting during Liquid Addition

Rewetting of the granule surface during granulation is incorporated in the model in the $\frac{dl_{e}}{dt}$ term. The rewetting depends on the volume of granule in comparison with the total volume of granule present in the system.

(18) $$\begin{array}{c} {\left.\frac{dl_{e}}{dt}\right|}_{rewetting}=\frac{{\dot{Q}}_{spray}\times v}{\sum_{s_{1}}\sum_{s_{2}}\sum_{p}Fv} \end{array}$$

#### 2.2.4. Granule Surface Growth Due to Solid-Spread Nuclei

As the pore volume of solid-spread nuclei are larger than that of the immersion nuclei, they are not directly added to the PBM equation. Instead the rate of change of number density of solid-spread nuclei ($F_{ss}$) is tracked using a differential equation. When two solid-spread nuclei aggregate, the resulting volume is divided proportionally between the $F_{ss}$ and *F* distribution using two class PBM approaches discussed by Jeong and Choi [33]. The depletion of solid-spread nuclei ($F_{ss}$) due to nuclei–nuclei aggregation and nuclei–granule surface growth is formulated as shown in Jeong and Choi [33] (Equation (19)).

(19) $$\begin{array}{c} {\left.\frac{ds_{1}}{dt}\right|}_{surfacegrowth}=k_{sg}v^{2/3} \end{array}$$

**Figure 2:**
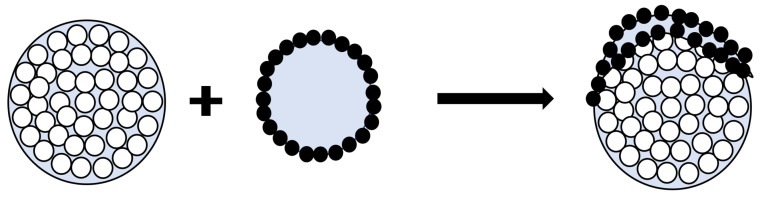
Solid-spread nuclei interaction with granule resulting in surface growth.

Planchette et al. [34] studied the transition of liquid marble onto solid surfaces. They studied three mechanisms involved when solid-spread nuclei collide on a surface. The drop extension of the solid-spread nuclei is related to the impact velocity as $\left(D_{max}-D\right)/D≊0.12\sqrt{(}We)$, where $D_{max}$ is the diameter of the disk shape the solid-spread nuclei takes before rupture, *D* is the diameter of the solid-spread nuclei (=$d_{d}+2d_{p}$), and We is the Weber number during collision. This equation gives the minimum size (=$f(D_{max})$) of the granule upon which, when the solid-spread nuclei collide, the impact results in the surface growth of $s_{1}$ particles, as shown in Figure 2.

#### 2.2.5. Hydrophilic Excipient Layering

Hydrophilic excipient layering is modeled as an increase in granule size as fine excipient powder particles adhere to the wet surfaces. The rate of increase in excipient volume of a granule ($\frac{ds_{2}}{dt}$) is assumed to be proportional to the granule surface area (Equation (5)) and the total mass of excipient powder left ($m_{s2}$) in the granulator as shown in the following equations.

(20) $$\begin{array}{c} \frac{ds_{2}}{dt}=\frac{k_{layer}\times a\times m_{s2}}{\rho_{s2}} \end{array}$$

It is modeled such that only the granules with surface wetness ($l_{e}\neq 0$) experience layering. As a result, the increase in consolidation has a secondary effect on layering. The depletion of excipient fines is given by Equation (21).

(21) $$\begin{array}{c} \frac{dm_{s2}}{dt}=\rho_{s2}\int_{0}^{s1}\int_{0}^{s2}\int_{p_{0}}^{p}F\frac{ds_{2}}{dt}ds_{1}ds_{2}dp \end{array}$$

#### 2.2.6. Particle Aggregation

**Figure 3:**
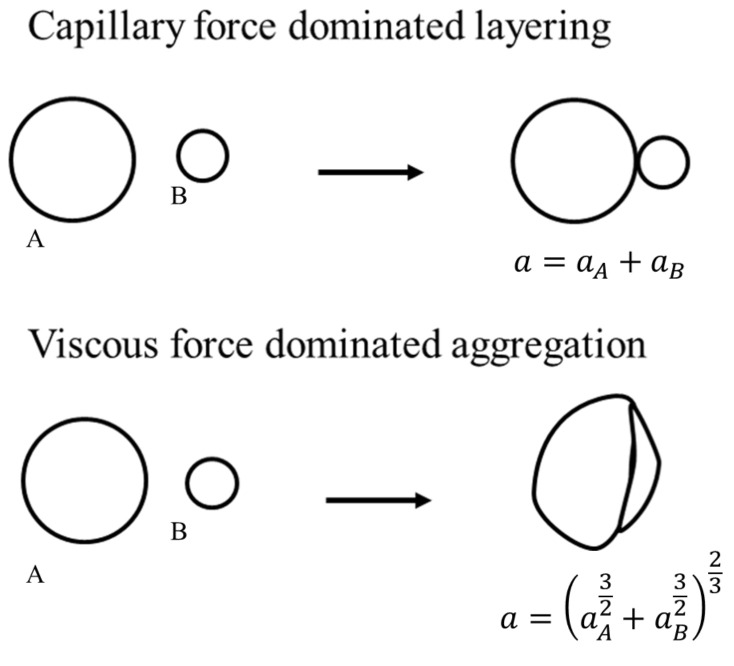
Representation of soft and hard granule growth.

Goodson et al. [16] developed a PBM framework for characterizing the granule based on three properties: (1) size (big or small), (2) liquid content (wet or dry), and (3) strength (hard or soft). Figure 3 shows the two extremes of granule interactions. Two granules of high strength, less deformability, and lower capillary number are assumed to conserve surface area upon collision (Type I) and two granules of low strength, high deformability, and higher capillary number are assumed to conserve pore volumes upon collision.

The solid volumes are conserved during the aggregation. The resulting aggregate solid volume is a sum of the two colliding particle solid volume. The pore volume and liquid volume undergo a different transformation rule. Depending on the deformability of the colliding particles, the final granule pore volume is an interpolation of the two extremes in Figure 3. The resulting pore volume can be written as,

(22) $$\begin{array}{c} a=\zeta \left(a_{A}^{\frac{3}{2}}+a_{B}^{\frac{3}{2}}\right)+\left(1-\zeta \right)\left(a_{A}+a_{B}\right),\;0<\zeta <1 \end{array}$$

(23) $$\begin{array}{c} p=\frac{a^{\frac{3}{2}}}{6\sqrt{\pi }}-s_{1}-s_{2}-l_{e} \end{array}$$

where ζ represents the relative softness or deformability of the granule (a function of coefficient of restitution, $\zeta =1-e_{coag}$ from Equation (36)). This internal coordinate representation was shown to provide differences in critical properties such as granule porosity, despite the similar granule size predictions [16].

The rate of particle aggregation is calculated as shown below.

(24) $$\begin{array}{cc} R_{agg}\left(s_{1},s_{2},p,t\right) & =R_{agg}^{form}\left(s_{1},s_{2},p,t\right)-R_{agg}^{dep}\left(s_{1},s_{2},p,t\right) \end{array}$$

where $R_{agg}^{form}$ and $R_{agg}^{dep}$ are the rates of formation of larger particles (Equation (26)) and rate of depletion of smaller particles (Equation (27))), respectively.

(25) $$\begin{array}{cc} R_{agg}^{form}\left(s_{1},s_{2},p,t\right) & =\frac{1}{2}\int_{0}^{s_{1}}\int_{0}^{s_{2}}\int_{p_{0}}^{p}\beta \left(s_{1}-s_{1}^{{}^{\prime }},s_{2}-s_{2}^{{}^{\prime }},p-p^{{}^{\prime }},s_{1}^{{}^{\prime }},s_{2}^{{}^{\prime }},p^{{}^{\prime }}\right)\times \\ & F\left(s_{1}-s_{1}^{{}^{\prime }},s_{2}-s_{2}^{{}^{\prime }},p-p^{{}^{\prime }},t\right)F\left(s_{1}^{{}^{\prime }},s_{2}^{{}^{\prime }},p^{{}^{\prime }},t\right)ds_{1}^{{}^{\prime }}ds_{2}^{{}^{\prime }}dp^{{}^{\prime }} \end{array}$$

(26) $$\begin{array}{cc} R_{agg}^{dep}\left(s_{1},s_{2},p,t\right) & =F\left(s_{1},s_{2},p,t\right)\int_{0}^{s_{1,max}-s_{1}}\int_{0}^{s_{2,max}-s_{2}}\int_{p_{0}}^{p_{max}-p}\\ & \beta \left(s_{1},s_{1}^{{}^{\prime }},s_{2},s_{2}^{{}^{\prime }},p,p^{{}^{\prime }}\right)\times F\left(s_{1}^{{}^{\prime }},s_{2}^{{}^{\prime }},p^{{}^{\prime }},t\right)ds_{1}ds_{2}dp \end{array}$$

The rate of aggregation $R_{agg,l_{i}}$ and $R_{agg,l_{e}}$ in Equations (2) and (3) are the rates at which the internal and external liquid volumes are transferred between particles due to aggregation. Similar to the formation and depletion of what was discussed above, these rates can be calculated as shown in Equations (28) and (29).

(27) $$\begin{array}{cc} R_{agg,l_{i}}\left(s_{1},s_{2},p,t\right) & =\frac{1}{2}\int_{0}^{s_{1}}\int_{0}^{s_{2}}\int_{p_{0}}^{p}\beta \left(s_{1}-s_{1}^{{}^{\prime }},s_{2}-s_{2}^{{}^{\prime }},p-p^{{}^{\prime }},s_{1}^{{}^{\prime }},s_{2}^{{}^{\prime }},p^{{}^{\prime }}\right)\times \\ & F\left(s_{1}-s_{1}^{{}^{\prime }},s_{2}-s_{2}^{{}^{\prime }},p-p^{{}^{\prime }},t\right)F\left(s_{1}^{{}^{\prime }},s_{2}^{{}^{\prime }},p^{{}^{\prime }},t\right)\\ & \left(l_{i}\left(s_{1}-s_{1}^{{}^{\prime }},s_{2}-s_{2}^{{}^{\prime }},p-p^{{}^{\prime }},t\right)+l_{i}\left(s_{1}^{{}^{\prime }},s_{2}^{{}^{\prime }},p^{{}^{\prime }},t\right)+l_{e\to i}\right)ds_{1}^{{}^{\prime }}ds_{2}^{{}^{\prime }}dp^{{}^{\prime }}\\ & -L_{i}\left(s_{1},s_{2},p,t\right)\int_{0}^{s_{1,max}-s_{1}}\int_{0}^{s_{2,max}-s_{2}}\int_{p_{0}}^{p_{max}-p}\\ & \beta \left(s_{1},s_{1}^{{}^{\prime }},s_{2},s_{2}^{{}^{\prime }},p,p^{{}^{\prime }}\right)\times F\left(s_{1}^{{}^{\prime }},s_{2}^{{}^{\prime }},p^{{}^{\prime }},t\right)ds_{1}ds_{2}dp \end{array}$$

(28) $$\begin{array}{cc} R_{agg,l_{e}}\left(s_{1},s_{2},p,t\right) & =\frac{1}{2}\int_{0}^{s_{1}}\int_{0}^{s_{2}}\int_{p_{0}}^{p}\beta \left(s_{1}-s_{1}^{{}^{\prime }},s_{2}-s_{2}^{{}^{\prime }},p-p^{{}^{\prime }},s_{1}^{{}^{\prime }},s_{2}^{{}^{\prime }},p^{{}^{\prime }}\right)\times \\ & F\left(s_{1}-s_{1}^{{}^{\prime }},s_{2}-s_{2}^{{}^{\prime }},p-p^{{}^{\prime }},t\right)F\left(s_{1}^{{}^{\prime }},s_{2}^{{}^{\prime }},p^{{}^{\prime }},t\right)\\ & \left(l_{e}\left(s_{1}-s_{1}^{{}^{\prime }},s_{2}-s_{2}^{{}^{\prime }},p-p^{{}^{\prime }},t\right)+l_{e}\left(s_{1}^{{}^{\prime }},s_{2}^{{}^{\prime }},p^{{}^{\prime }},t\right)-l_{e\to i}\right)ds_{1}^{{}^{\prime }}ds_{2}^{{}^{\prime }}dp^{{}^{\prime }}\\ & -L_{e}\left(s_{1},s_{2},p,t\right)\int_{0}^{s_{1,max}-s_{1}}\int_{0}^{s_{2,max}-s_{2}}\int_{p_{0}}^{p_{max}-p}\\ & \beta \left(s_{1},s_{1}^{{}^{\prime }},s_{2},s_{2}^{{}^{\prime }},p,p^{{}^{\prime }}\right)\times F\left(s_{1}^{{}^{\prime }},s_{2}^{{}^{\prime }},p^{{}^{\prime }},t\right)ds_{1}ds_{2}dp \end{array}$$

The transfer of external liquid volume to internal liquid volume due to aggregation (represented as $l_{e\to i}$ in Equations (28) and (29)) can be computed as discussed by Braumann et al. [31].

(29) $$\begin{array}{cc} l_{e\to i} & ={\left[l_{e}\left(s_{1}-s_{1}^{{}^{\prime }},s_{2}-s_{2}^{{}^{\prime }},p-p^{{}^{\prime }},t\right)l_{e}\left(s_{1}^{{}^{\prime }},s_{2}^{{}^{\prime }},p^{{}^{\prime }},t\right)\right]}^{\frac{1}{2}} \end{array}$$

(30) $$\begin{array}{cc} \; & \times {\left[1-\sqrt{1-{\left(\frac{\sqrt[{3}]{v\left(s_{1}-s_{1}^{{}^{\prime }},s_{2}-s_{2}^{{}^{\prime }},p-p^{{}^{\prime }},t\right)-l_{e}\left(s_{1}-s_{1}^{{}^{\prime }},s_{2}-s_{2}^{{}^{\prime }},p-p^{{}^{\prime }},t\right)}}{\sqrt[{3}]{v(s_{1}-s_{1}^{{}^{\prime }},s_{2}-s_{2}^{{}^{\prime }},p-p^{{}^{\prime }},t)}+\sqrt[{3}]{v(s_{1}^{{}^{\prime }},s_{2}^{{}^{\prime }},p^{{}^{\prime }},t)}}\right)}^{2}}\right]}^{\frac{1}{2}}\\ \; & \times {\left[1-\sqrt{1-{\left(\frac{\sqrt[{3}]{v\left(s_{1}^{{}^{\prime }},s_{2}^{{}^{\prime }},p^{{}^{\prime }},t\right)-l_{e}\left(s_{1}^{{}^{\prime }},s_{2}^{{}^{\prime }},p^{{}^{\prime }},t\right)}}{\sqrt[{3}]{v(s_{1}-s_{1}^{{}^{\prime }},s_{2}-s_{2}^{{}^{\prime }},p-p^{{}^{\prime }},t)}+\sqrt[{3}]{v(s_{1}^{{}^{\prime }},s_{2}^{{}^{\prime }},p^{{}^{\prime }},t)}}\right)}^{2}}\right]}^{\frac{1}{2}} \end{array}$$

The aggregation kernel β depends on the properties of the colliding particle **A**($s_{1},s_{2},p$) and **B**($s_{1}^{{}^{\prime }},s_{2}^{{}^{\prime }},p^{{}^{\prime }}$) (as shown in Figure 3). $\beta \left(A,B\right)=\beta_{0}\beta^{*}A,B$. $\beta_{0}$ is independent of the colliding particle properties and is an optimized parameter in this work. $\beta^{*}$ is the efficiency of particle collision which can be determined based on the following model proposed by Balakin et al. [35]. The model accounts for both capillary and viscous forces during particle collision. The efficiency is determined as a ratio of the total work of forces within the liquid bridge to the kinetic energy of the particle.

(31) $$\begin{array}{c} \beta^{*}=\frac{W_{c}+W_{d}}{E_{k}}\Psi \end{array}$$

where $W_{c}$ and $W_{d}$ are the work of the capillary and dissipative forces, respectively. $E_{k}$ is the kinetic energy calculated from the mean relative velocity ($v_{r}$), mass (*m*), and coefficient of restitution (*e*, Equation (37)) of particle before collision, as shown in Equation (32). The relative velocity is calculated from the granular temperature (Θ), as given by Equation (33).

(32) $$\begin{array}{cc} E_{k} & =\frac{1}{2}me^{2}v_{r}^{2} \end{array}$$

(33) $$\begin{array}{cc} v_{r} & =\frac{3}{2}\sqrt{\pi \Theta } \end{array}$$

(34) $$\begin{array}{cc} \Theta & =\frac{\left(5\pi /96\right)\gamma^{2}d^{2}}{12\phi_{p}^{2}\left(1-{\left(\frac{\phi_{p}}{1-\phi_{p}}\right)}^{2}\right)} \end{array}$$

where $\phi_{p}$ is the volume fraction of particle in the granulator, γ is the shear rate, and *d* is the granule diameter. The work of dissipative force is calculated using the following equation.

(35) $$\begin{array}{c} W_{d}=\frac{3\pi \mu {\tilde{d}}^{2}e_{coag}v_{r}}{4}ln\left(\frac{h}{h_{a}}\right) \end{array}$$

where $\tilde{d}$ is the harmonic mean diameter of the colliding two particles and μ is the viscosity of the binder. *h* is the binder layer thickness calculated from the external liquid content ($l_{e}$) and $h_{a}$ represents the granule surface asperities. The resulting aggregated particle coefficient of restitution ($e_{coag}$) is represented as a function of coefficient of restitution of the constituent material properties by Braumann et al. [31] by the following equations:

(36) $$\begin{array}{c} e_{coag}=\sqrt{e_{A}e_{B}} \end{array}$$

(37) $$\begin{array}{c} e_{i}=\frac{\sum_{\alpha }e_{\alpha }m_{\alpha }}{\sum_{\alpha }m_{\alpha }},i\in \left\{A,B\right\} \end{array}$$

where $\alpha \in \{s_{1},s_{2},p\}$, *m* is the mass of colliding granule and *e* is the ratio of rebound energy to impact energy. It takes a value between 0 (totally plastic impact) and 1 (totally elastic impact). *e* for pore is assumed to be 0.

**Figure 4:**
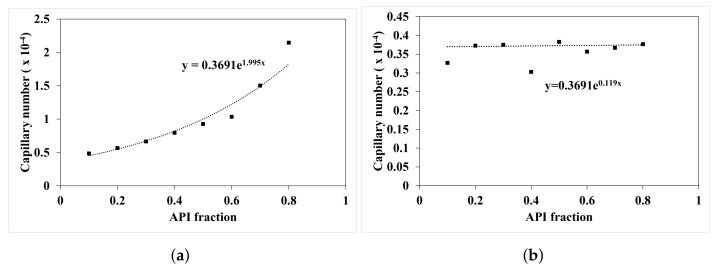
Change in capillary numbers with increase in API fraction. (**a**) Ibuprofen and MCC101 formulation, (**b**) Acetaminophen (APAP) and MCC101 formulation.

The work of capillary force ($W_{c}$) is experimentally determined from the regime map analysis carried out Muthancheri and Ramachandran [22]. Figure 4 plots the capillary number which is the ratio between capillary force and viscous force as a function of API fraction. The equation determined from the experiment analysis is used to provide a composition dependent work of capillary force in the model.

#### 2.2.7. Compaction

Compaction of granules occur during collision and result in porosity reduction of the granules.

(38) $$\begin{array}{c} \Delta \epsilon =\left\{\begin{array}{cc} k_{con}U\left(\epsilon -\epsilon_{min}\right) & \mathrm{if}\;\epsilon -\Delta \epsilon \geq \epsilon_{min}\\ 0 & \mathrm{otherwise} \end{array}\right. \end{array}$$

where $k_{con}$ is the consolidation rate constant, *U* is the particle collision velocity, and $\epsilon_{min}$ is the minimum porosity. Two conditions are modeled in this study. A non-squeeze case: if no internal liquid is transferred to the external surface. In this case, there is only pore volume reduction due to consolidation (Equation (39)). The next scenario is a squeeze case. Some liquid is transferred from internal to external liquid volume (Equation (40)). This occurs if the porosity after consolidation is smaller than a critical porosity. In this scenario, the pore volume is completely occupied by internal liquid volume ($l_{i}=p$).

(39) $$\begin{array}{c} \frac{dp}{dt}=-\left(\frac{1}{1-(\epsilon -\Delta \epsilon )}\left(s_{1}+s_{2}+l_{e}\right)-v\right) \end{array}$$

(40) $$\begin{array}{c} \frac{dp}{dt}=-\left(\left(1+\left(\epsilon -\Delta \epsilon \right)\right)\left(s_{1}+s_{2}+l_{e}\right)+\left(\epsilon -\Delta \epsilon \right)l_{i}-v\right) \end{array}$$

#### 2.2.8. Particle Breakage

The particle breakage occurs when a large particle disintegrates into two or more daughter (or smaller) particles. The net particle breakage rate is modeled using the method described in Barrasso and Ramachandran [32]. The details of the model are provided in the Appendix A.

### 2.3. Hybrid Modeling

**Figure 5:**
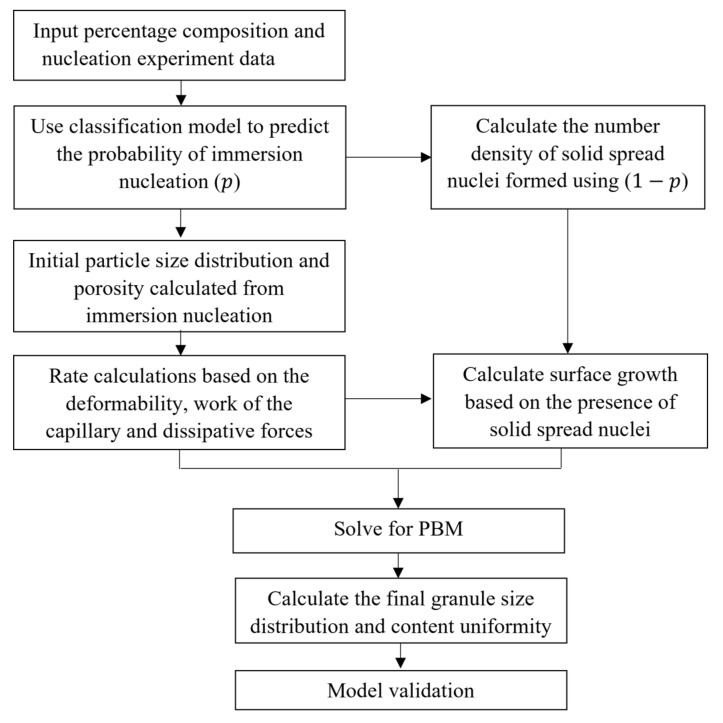
Hybrid modeling framework.

Figure 5 depicts the hybrid modeling framework. The data-driven classification model presented in the previous literature [30] is used to predict the probability of nucleation (*P*). Based on that probability, the number of immersion nuclei and solid-spread nuclei is determined. The porosity and size of the initial nuclei effects the granule growth through the aggregation kernel and API surface growth discussed in Section 2.2.

### 2.4. Numerical Solution

The ordinary differential equations (ODE) obtained after discretization for different particle size combinations are integrated simultaneously using the first-order explicit Euler integration technique, which is popularly used to solve PBMs [5,9,36,37,38]. The numerical stability of a PBM is complex due to the presence of multiple dimensions and the inherent possibility of instability involved with the time-step of the integration. The integration time-step was thus chosen, such that the rate of particles leaving a particular size class (bin) is not higher than the number of particles in that size class at any time-step based on the Courant–Friedrichs–Lewy (CFL) condition [8,39]. The partial derivatives with respect to internal coordinate volume ($u\in (s_{1},s_{2},p)$) and time (*t*) were discretized using a non-linear grid ($u_{i}=u\times {\left(4\right)}^{i-1}$). Here, *i* represents the bin number in one dimension, and *u* indicates the volume of particle in the smallest bin. The smallest particle size is 31.5 μm, and the largest particle size is 6000 μm. There is a total of 20 bins or grid points. A cell average technique discussed by Chaudhury et al. [7] is utilized in this study to distribute the particles that are formed in the intermediate range of two bins, into the adjacent bins. The computations were performed in MATLAB 2020a on an Intel(R) Core(TM) i7-8700 CPU (3.20 GHz) with 16 GB RAM.

### 2.5. Sensitivity Analysis

A sensitivity study was performed to investigate the effect of adjustable parameters on the simulation results. The optimized values of parameters were perturbed from −20% to 20% with a step size of 10%, and the results were compared to the base value. The sensitivity is measured using Equation (41).

(41) $$\begin{array}{c} Sensitivity=\left|\frac{Y_{0}^{j}\left(t\right)-Y_{i}^{j}\left(t\right)}{Y_{0}^{j}\left(t\right)}\right| \end{array}$$

where $Y_{i}^{j}\left(t\right)$ is the value of granule property of interest in the *i*th perturbation of the *j*th parameter and $Y_{0}^{j}\left(t\right)$ is the base value for the *j*th parameter.

**Figure 6:**
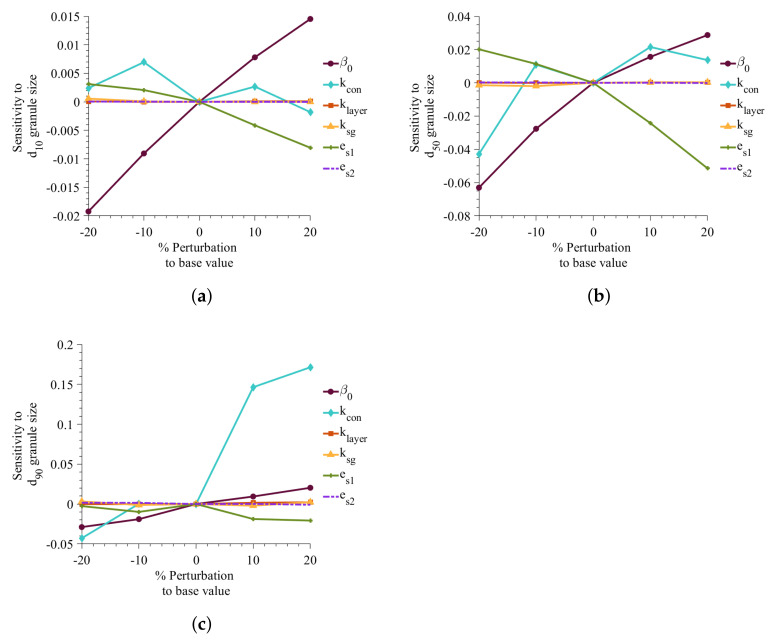
Effect of changes in adjustable parameter values on d_10_, d_50_, and d_90_. Sensitivity to (**a**) d_10_, (**b**) d_50_, (**c**) d_90_.

Figure 6 illustrates that the sensitivity of parameters on d_10_, d_50_, and d_90_ simulation. It shows that growth parameters ($k_{layer}$ and $k_{sg}$) are much less sensitive than the aggregation and consolidation parameters when the variables are perturbed ±20%. The average diameter is found to be highly sensitivity toward the coefficient of restitution of API ($e_{s1}$). The study shows a decrease in average diameter with an increase in $e_{s1}$. A decrease in $e_{s1}$ indicates that the API is very deformable, resulting in smaller average granule size. The aggregation rate constant, $\beta_{0}$, has a positive impact on the granule size, showing an increase in the rate constant increasing the average granule size. The consolidation rate equation has a negative term (Equations (39) and (40)), which means the increase in $k_{con}$ results in a decrease in consolidation rate. In Figure 6c, it can be seen that a decrease in consolidation rate to 20% results in larger granules.

**Figure 7:**
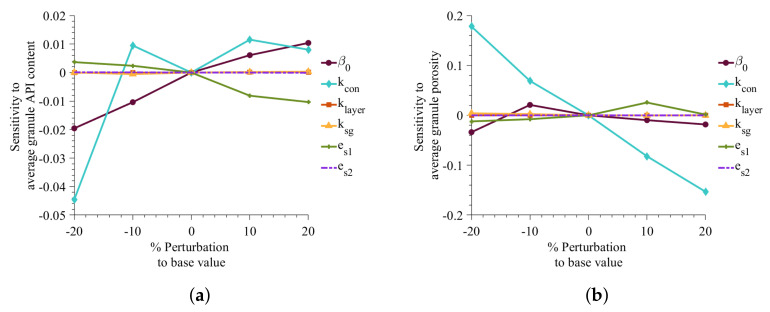
Effect of changes in adjustable parameter values on porosity and API content. (**a**) Sensitivity to average API content, (**b**) Sensitivity to granule average porosity.

Similarly, Figure 7 shows the effect of the adjustable parameters on the average porosity and API content of the granules. The sensitivity of parameters to granule API content is similar to that of the granule size. Aggregation rate constant, coefficient of restitution, and consolidate rate were found to be most significant in impacting the granule API content. A decrease in $e_{s1}$ results in a decrease in aggregation rate and thus results in granules with less $s_{1}$ or API content. Average porosity of granule is most impacted by the consolidation rate. It can be seen that with increase in consolidation (or decrease in consolidation rate constant) the average granule porosity decreases.

## 3. Results and Discussions

### 3.1. Optimization and Parameter Estimation

Experimental data from Muthancheri and Ramachandran [22] is used to estimate the unknown parameters in the model. The cumulative granule size fraction and content uniformity measurements were used for the estimation. For the multi-objective optimization for minimizing the error for both size and content uniformity, a Pareto optimal solution techniques was used. The method provides an optimal solution when one objective function cannot decrease without increasing the other objective function [40].

The tuned parameters are aggregation constant ($\beta_{0}$), coefficient of restitution of API ($e_{s1}$) and excipient ($e_{s2}$), consolidation rate constant ($k_{con}$), excipient layering rate constant ($k_{layer}$), and rate of surface growth ($k_{sg}$). Out of the six experimental runs, four were used for parameter estimation, and two were used to validate the calibrated model. The optimized values of the variables are provided in the Table 2.

### 3.2. Model Training and Validation

**Figure 8:**
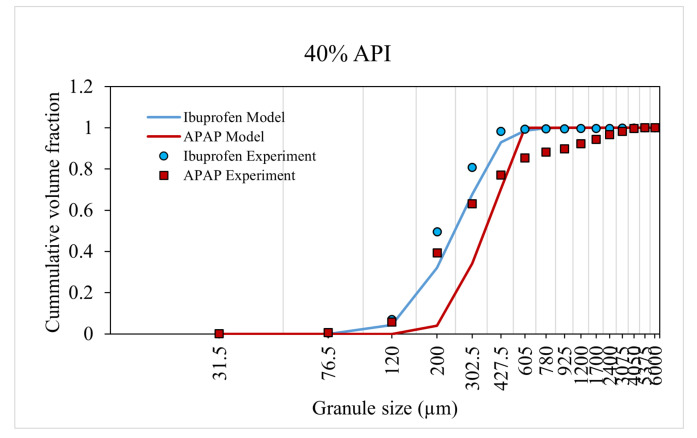
Model prediction for 40% API formulation.

**Figure 9:**
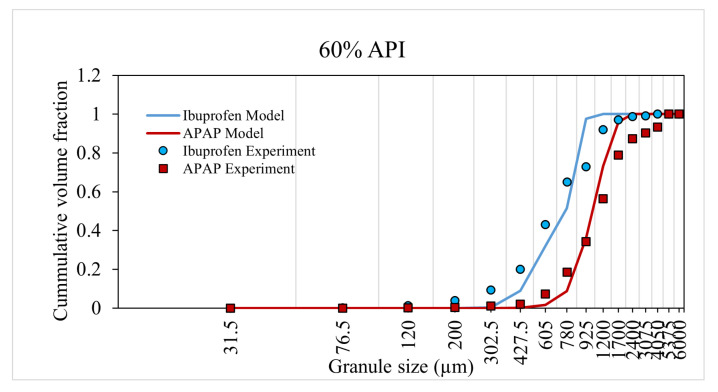
Model prediction for 60% API formulation.

**Figure 10:**
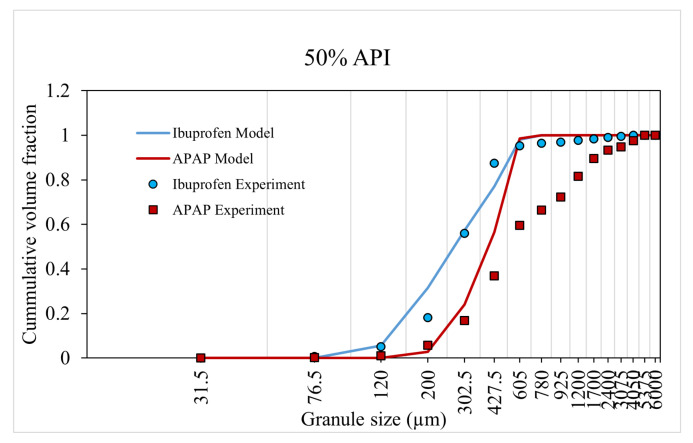
Model validation using 50% API formulation.

The model was trained and validated using the experiment data from Muthancheri and Ramachandran [22]. In the aforementioned study, two formulations were considered such that there exists a wettability differential between the two components in a formulation. The two cases were (1) ibuprofen-USP and micro-crystalline cellulose and (2) micronized acetaminophen (APAP) and micro-crystalline cellulose. The 40% and 60% cumulative size distributions of formulations were used for model training (Figure 8 and Figure 9). The 50% cumulative size distributions of formulations were used for model validation (Figure 10). This validation helps to concur the assumptions and theories considered in the model. The predicted values had a strong relationship with the experimental values. The overall accuracy of the model was estimated to be 0.89 (Overall accuracy = 1 − sum of square error).

An increase in API percentage composition from 40% to 60% experimentally resulted in an overall increase in granule size or a shift in the distribution curve to the right for both case-I and -II formulation. The model was able to accurately predict this increase in granule size (and distribution) at a high API percentage composition. This dependency of the API composition was reflected in the model through the composition dependent aggregation kernel and nucleation probability.

**Figure 11:**
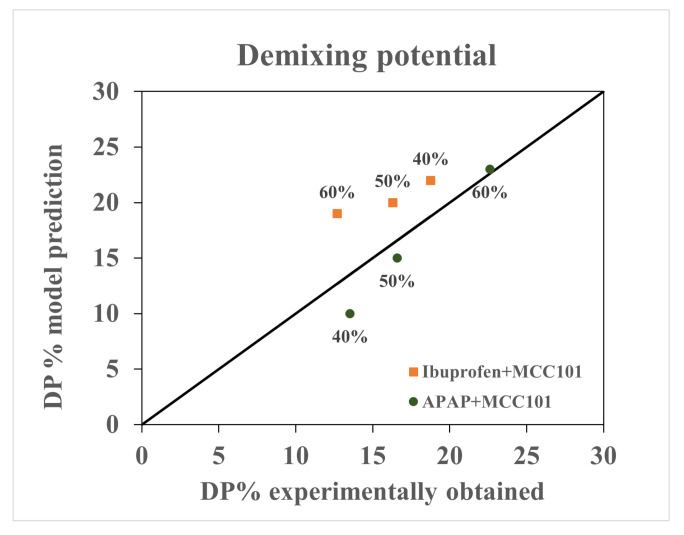
Comparison between model prediction and experimentally obtained de-mixing potential.

The content uniformity of the granules were evaluated using the demixing potential (DP) introduced by Thiel and Nguyen [41] to quantify the distribution of a solid component as a function of particle size. DP can be calculated using the following equation:

(42) $$\begin{array}{c} \mathrm{DP}\%=\frac{100}{\bar{x}}\sqrt{\sum w{\left(x-\bar{x}\right)}^{2}} \end{array}$$

where *x* is the API content in a particular size range, $\bar{x}$ is the average API content, and *w* is the weight fraction of granule in each size range. The quantity is similar to the relative standard deviation used for non-uniformity in mixing by Oka et al. [42]. The larger the value of de-mixing potential, the larger the extent of deviation from the mean of the API across granule size classes. Figure 11 shows the ability of the presented hybrid-modeling framework to predict the change in de-mixing potential with an increase in percentage API. The model predicted a decrease in the extend of de-mixing with increase in percentage API for ibuprofen formulation and an increase in the extent of de-mixing with increase in percentage API for APAP formulation.

### 3.3. Model Applications

#### 3.3.1. Effect of Change in Formulation on Dynamic Granule Formation

Muthancheri and Ramachandran [22] studied the effect of increase in hydrophobic API percentage on the granule size. It was shown that in an insoluble system (all constituent materials were insoluble in the binder liquid), an increase in hydrophobic component results in an overall increase in granule size.

**Figure 12:**
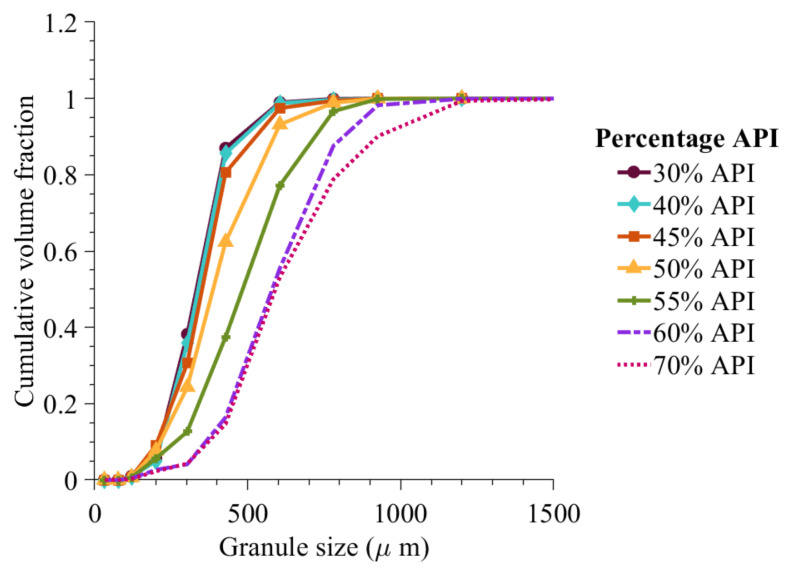
Cumulative volume fraction prediction with change in percentage composition of API and 0.6 liquid-to-solid ratio.

Figure 12 shows that when the percentage API is varied from 30% to 60% the average particle size was found to increase. Here, the liquid-to-solid ratio was kept the same as that during the model optimization (0.6 liquid-to-solid ratio).

**Figure 13:**
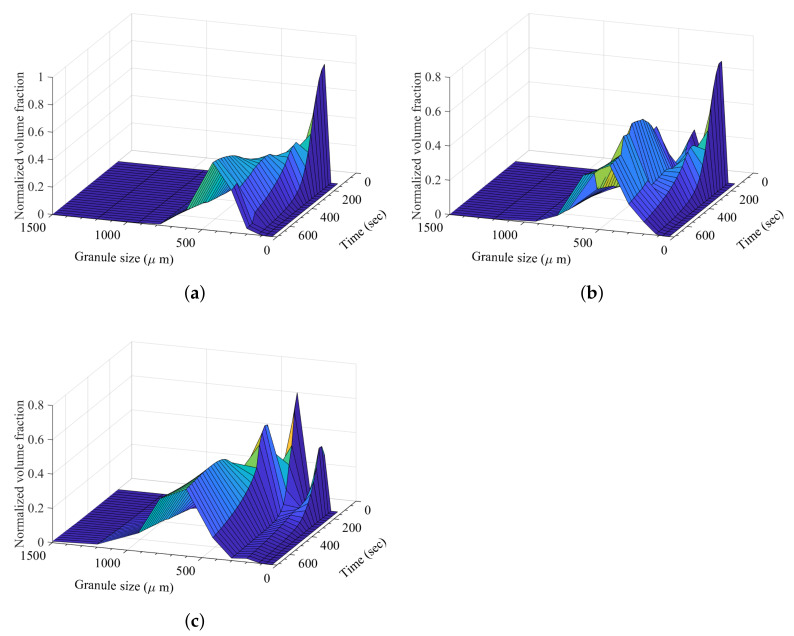
Granule size distribution with increase in granulation time at a varying degree of hydrophobic content (ibuprofen). (**a**) Granule size distribution with increase in granulation time at 40% hydrophobic component (ibuprofen), (**b**) granule size distribution with increase in granulation time at 50% hydrophobic component (ibuprofen), and (**c**) granule size distribution with increase in granulation time at 60% hydrophobic component (ibuprofen).

The effect of formulation or hydrophobicity in the dynamic granule formation can be observed in Figure 13. At 40% hydrophobic content, the probability of solid-spread nuclei formation was very low, resulting in a high initial immersion nuclei and consequent granule formation. At 50% hydrophobic component, the probability of solid-spread nuclei is modeled to be 0.67 (calculated from the classification model [30]). This resulted in a small secondary peak at the start of liquid addition (Figure 13b). At a high hydrophobic content of 60%, the high probability of solid-spread nuclei formation led to an increase in granule growth and larger final granules (Figure 13c).

**Figure 14:**
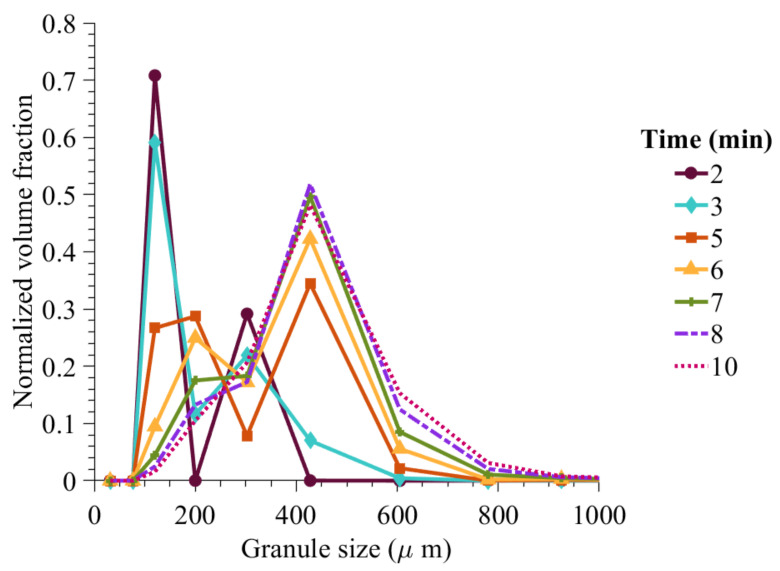
Model predicted granule size distribution with an increase in wet massing time (50% API content).

At 50% composition of ibuprofen in the formulation made of ibuprofen and MCC-101, the classification model predicted a probability of immersion nucleation to be 0.67. This scenario is further evaluated in Figure 14 in terms of dynamic evolution of granule size as granulation progress. It can be seen that due to wet massing, the solid-spread nuclei combine together to form larger granules and then combine with the immersion nuclei to form a wider distribution of granules.

#### 3.3.2. Effect of Change in Formulation on Granule API Content

**Figure 15:**
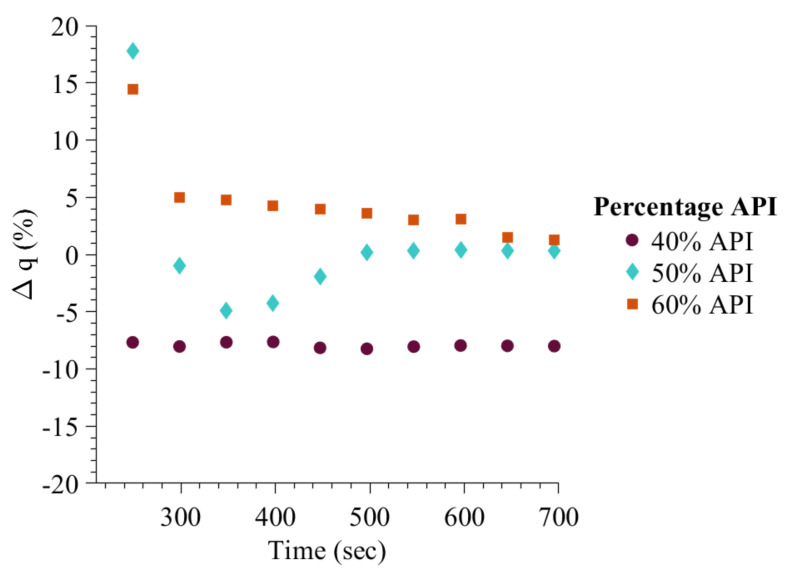
Model predicted granule API content with increase in wet massing time.

Muthancheri and Ramachandran [22] study showed that as the API content increased from 40% to 60%, the granule content non-uniformity decreased. Figure 15 shows a similar trend. As the hydrophobic API content increased from low to high, the Δq, which is the difference between the powder blend API content and average granule API content, became near zero. Smaller granules of high API content were also found during the experiment study, validating the high API content prediction at the start of granulation process.

#### 3.3.3. Effect of Change in Formulation on Average Granule Porosity

**Figure 16:**
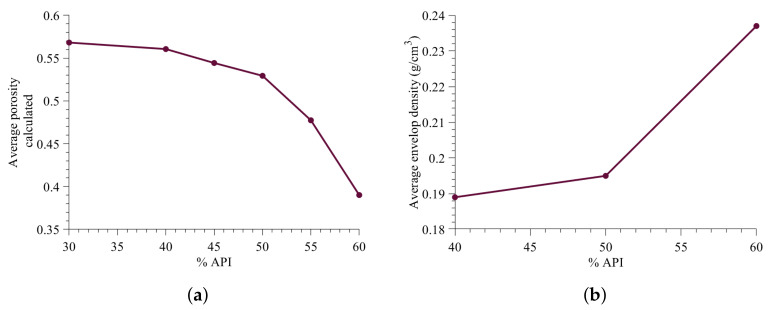
Change in granule micro-structure with increase in API content. (**a**) Model predicted average porosity, (**b**) Experimental envelop density.

Next, the average granule porosity prediction with change in the percentage API is evaluated. The average porosity prediction is within the porosity range reported previously in the high shear wet granulation model study [43]. The envelop density of the granules obtained in the Muthancheri and Ramachandran [22] was carried out to verify the trend of porosity predicted in Figure 16a.

The envelop density (Figure 16b) was measured using a graphite powder quasi-fluid, known as Dryflo (Micromeritics, Norcross, GA, USA) [44]. The porosity of granule is inversely related to the envelop density. Figure 16b shows the influence of increase in API percentage on the envelop density. Based on the envelop density trend, it is concluded that the decrease in average porosity predicted using the developed model is fairly accurate.

#### 3.3.4. Effect of Change in Formulation on Average Liquid Fraction

**Figure 17:**
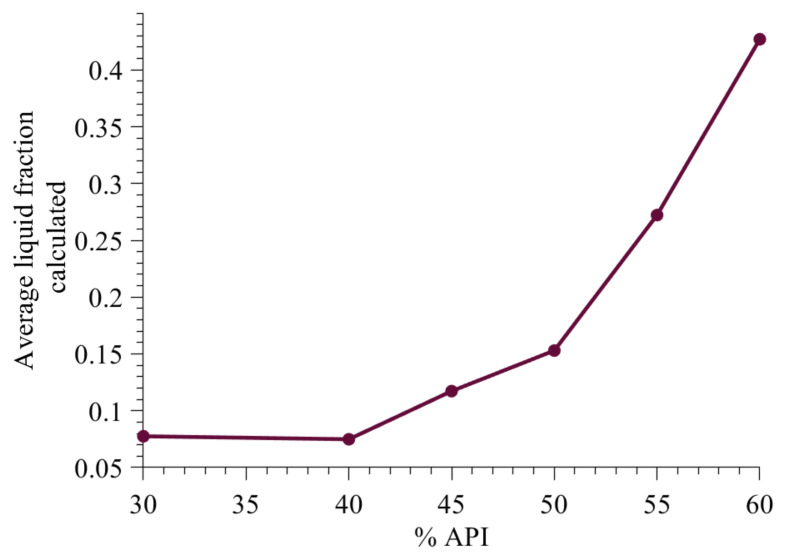
Model predicted average liquid fraction.

The change in average liquid fraction with an increase in API percentage is shown in Figure 17. The range of liquid fraction is similar to that reported in Chaudhury and Ramachandran [43]. The increase in liquid content is primarily due to increase in solid-spread nucleation, which results in surface growth. Surface growth of solid-spread nuclei increases the API content as well as liquid content on the granules. The liquid contant also increases due to decrease in hydrophilic excipient. Hydrophilic excipient reduced the available liquid due to the transfer of liquid from the external surface to inside the particles.

## 4. Conclusions

In this study, a hybrid model (Random Forest-PBM) is developed to describe the bi-component high shear wet granulation process. The model incorporates immersion and solid-spread nucleation based on the change in percentage API. The probability of each nucleation mechanism to occur, for a given formulation, is obtained from the random forest model. The probability is incorporated into the PBM framework such that the rate equations are impacted by the availability of immersion and solid-spread nuclei. It was found that the aggregation and consolidation rate are more sensitive to the granule critical quality attribute predictions. The model predictions are qualitatively in agreement with profiles obtained in the literature [22,43]. The discussed methodology and presented model could be used for predicting various aspects of the granulation process and controlling the transient behavior during the process. As an example, we have provided the average particle size, porosity, liquid content, and demixing-potential of the granules with change in API percentage. The developed model is an improvement to the existing mechanistic modeling framework, such that it incorporates the effect of hydrophobicity to track the granule critical quality attributes.

## Figures and Tables

**Table 1:** Variables in the PBM to describe granule.

Description	Notation
Independent variables	
API solid volume	$s_{1}$
Excipient solid volume	$s_{2}$
External liquid volume	$l_{e}$
Internal liquid volume	$l_{i}$
Pore volume	*p*
Dependent variables	
Total granule volume	*v*
Surface area	*a*
Porosity	ϵ
Content uniformity	*q*

**Table 2:** Optimized parameters.

Parameters	Notation	Value
Aggregation rate constant	$\beta_{0}$	$4.34\times 10^{-10}$
Ibuprofen coefficient of restitution	$e_{s1,ibu}$	0.162
APAP coefficient of restitution	$e_{s1,apap}$	0.103
MCC101 coefficient of restitution	$e_{s2}$	0.07
Consolidation rate constant	$k_{con}$	$1.67\times 10^{-3}$
Excipient layering rate constant	$k_{layer}$	$2.01\times 10^{-8}$
Surface growth rate constant	$k_{sg}$	$4.59\times 10^{-11}$

## Data Availability

Not applicable.

## Appendix A

Equation (A1) represents the breakage rate equation.

(A1) $$\begin{array}{cc} R_{brk}\left(s_{1},s_{2},p,t\right) & =R_{brk}^{form}\left(s_{1},s_{2},p,t\right)-R_{brk}^{dep}\left(s_{1},s_{2},p,t\right) \end{array}$$

where $R_{brk}^{form}$ and $R_{brk}^{dep}$ are the rates of formation of smaller particles and rate of depletion of larger particles.

(A2) $$\begin{array}{cc} R_{brk}^{form}\left(s_{1},s_{2},p,t\right) & =\int_{s_{1}}^{s_{1,max}}\int_{s_{2}}^{s_{2,max}}\int_{p}^{p_{max}}b\left(s_{1},s_{2},p,s_{1}^{{}^{\prime }},s_{2}^{{}^{\prime }},p^{{}^{\prime }}\right)K_{brk}\left(s_{1},s_{2},p\right)\times \\ & F\left(s_{1}^{{}^{\prime }},s_{2}^{{}^{\prime }},p^{{}^{\prime }},t\right)ds_{1}^{{}^{\prime }}ds_{2}^{{}^{\prime }}dp^{{}^{\prime }} \end{array}$$

(A3) $$\begin{array}{cc} R_{brk}^{dep}\left(s_{1},s_{2},p,t\right) & =K_{brk}\left(s_{1},s_{2},p\right)F\left(s_{1},s_{2},p,t\right) \end{array}$$

where $K_{brk}$ is the breakage rate constant and *b* is the probability distribution function of daughter particles. For the purposes of this study, a uniform probability distribution was assumed for all possible daughter particles [32].
